# The Institutional Worker

**Published:** 1913-08-23

**Authors:** 


THE
### INSTITUTIONAL WORKER
**Being a Special Supplement to "The Hospital"**

### OUR BUREAU OF INFORMATION.

### Rules for Correspondents.

1. Every letter must be accompanied by the coupon to be cut from the back cover (inside page) of The Hospital, current issue, and must contain the name and address of the correspondent with pseudonym for publication if desired. Replies by post cannot be given, save under exceptional circumstances at the Editor's discretion.

2. Letters from Approved Homes in reply to special needs published in the Bureau should state terms and full particulars, and be sent prepaid under cover to the Editor of the Bureau with name written across coupon for identification.

3. Proprietors of Homes which have not yet been entered on the List of Approved Homes, but have spare accommodation likely to suit special needs, are invited to write for an application form for registration. The fee for registration, which includes two announcements of the Home in the Bureau and other privileges, is 10s.

4. All communications to be addressed to the Editor of The Hospital, 28 Southampton Street, Strand, London, W.C., and marked "Bureau of Information."

### INSTITUTIONAL FACTS AND FIGURES.

This week we publish the second selected answer to Question 2 for July, which was: If you keep your accounts in accordance with the Uniform System, describe fully your method of checking, sorting, analysing, and filing your accounts for payment. Give such particulars as will clearly indicate the manner in which these are passed through your analysis journal, and onwards through the ledger. Add your further remarks. The reply deals with the method of accounting at a large provincial hospital, and a comparison between this and the account, published last week, of the method employed at a London hospital indicates several points of difference worthy of note, the main one being that a bought day book or monthly journal is not kept, the ledger being posted direct from the analysis journal. Our contributor, in his concluding remarks, draws attention to possible inaccuracy in comparing the cost of maintenance during corresponding periods owing to variation in the amount of stock in hand at the close of the periods. At many institutions the purchases are so regulated that such variations are negligible; but occasions necessarily arise when the question of stock must be taken into consideration in accounting for a rise or fall in expenditure. Whilst our contributor's suggestion for dealing with the difficulty is not compatible with the laws of accountancy, the point he raises is an interesting one, and has, no doubt, engaged the attention of other hospital officers, whose remarks upon the subject and the general question of stock-taking are invited.

### ACCOUNT CHECKING AT A PROVINCIAL HOSPITAL.

### Second Selected Answer.

At this institution, where we keep our accounts in accordance with the "Uniform System," all goods are ordered and the invoices for same checked by either the general stores department or the dispensary and surgical instruments department.

#### How Invoices are Checked.

The process of dealing with the invoices consists of (1) checking the weight or quantities as stated on the invoice with the weight or quantities as entered by the person receiving same in the goods received book of the department at which the goods were received; (2) comparing the prices charged with the quotations, contracts, etc., or previous rates paid as the case may be; (3) the general checking of the invoice as to calculations, totals, etc.; (4) agreeing the invoice with the duplicate order in the official order book and entering on the duplicate order the date the invoice was passed and on the invoice the official order number; (5) entering the substance of the invoice in the various books of the department; (6) marking the invoice with the name of the account to which the amount is chargeable in accordance with the Uniform System; (7) the initialing of the invoice by the clerk entering and checking same; (8) the signing of the invoice by the head of the department.

#### Method of Filing.

The invoices are then passed to the financial department, where they are signed by the house governor and each month's invoices filed in alphabetical order and according to date, one file being used for the invoices of each department.

#### Entering the Analysis Book.

From these files the invoices are entered into the analysis of expenditure book, and at the same time the invoices are numbered consecutively and the invoice number used as a reference number in the analysis book. The analysis book folio is also entered on the invoice for reference purposes.

#### Posting the Ledger.

The amounts of the invoices are posted direct from the analysis of expenditure book to the credit of the various firms in the personal ledger.

The monthly and quarterly accounts are, of course, paid on the ledger balance after agreement with the monthly or quarterly statements received from the firms.

#### A Suggested Improvement.

Personally I consider the charging of invoices as far as possible to a departmental stores account, instead of direct to the sub-head of the income and expenditure account, and charging only the consumption at the end of each month to the sub-head, is a better method than charging direct the total of the invoice. By so doing your income and expenditure account shows on the expenditure side consumption instead of purchases, which is a much better figure for comparative purposes.—"Institutional."

---

### Refreshments for Out-patients.

Amongst the numerous outlets for the activities of a Ladies' Association is the provision of refreshments for out-patients at moderate prices. The long period of waiting in the out-patient department, which, unfortunately, cannot be avoided, is a source of much discomfort to the patients, and many of them, more especially those who come from a distance, suffer considerably through lack of food. Wherever this provision has been made it has proved a great boon, and at one institution with which we are acquainted, where the work is supervised by a Ladies' Association, the profit made helps to maintain a bed at a convalescent home.

### QUESTIONS for AUGUST.

AUGUST is a holiday-making time, and therefore the questions we are publishing for this month are of such a nature as to be easy to answer without recourse to reference books. Question 2 touches on a branch of philanthropy which is coming greatly to the fore, and practical workers in this field are invited to give their experience which cannot fail to be of considerable help to the many beginners whom this work is attracting. Question 1 should appeal to the secretariat of any institution, and their views, with the reasons for such views, will be welcome.

**Any Use for an Addressograph ?**

**1. Is it or is it not an advantage for a hospital to possess an addressograph? Give reasons, and if an advantage give particulars of cost.**

**Mothers' Dinners Menus.**

**2. Give a week's menu for dinners for mothers in connection with Schools for Mothers, with the daily cost for 20 people, bearing in mind the need for nourishing food and for strict economy.**

RULES.

The following rules must be observed:—

1. Contributions must be written on one side of the paper. They must bear the name and address of the sender and be accompanied by coupon to be cut from the back cover (inside page) of the current issue of THE HOSPITAL. A pseudonym must be chosen if the name is not to be published.

2. They must be addressed to the Editor of THE HOSPITAL, 28 & 29 Southampton Street, Strand, London, W.C., so as to reach him by the last day of the month for which the questions are set, and must be marked in the left-hand corner "Facts and Figures."

**A minimum payment of Five Shillings will be made for each published answer.**

### THE SICK and IN NEED.

### Homes for Epileptics.

EPILEPTIC women are provided for at the Chalfont Colony, Bucks, and cases are admitted, upon application to the National Society for Epileptics, Denison House, Vauxhall Bridge Road, S.W., for a weekly payment of 10s. At the Home for Epileptics, Maghull, near Liverpool, cases are received in the third division for 10s. 6d. per week; and at the Meath Home of Comfort for Epileptics, Westbrook, Godalming, women not over 35 years of age are eligible for admission upon a weekly payment of 12s. 6d. You should apply to all three institutions without delay, for the pressure upon their beds is very heavy, and the waiting period prolonged.—" J. N."

### Approved Home.

WE have pleasure in adding the following Home to our Approved List:

KENT.—The West Kent Nursing Home, Bridgen Lodge, Bexley Heath (eleven miles from London). Principal: Mrs. S. J. Weston. A well-equipped Home for medical, surgical, maternity, and rest-cure cases. The Home is situated 180 feet above sea-level, and the locality is well known as a health resort. Terms from £1 11s. 6d. to £6 6s., in accordance with accommodation and character of cases. Telephone: 179 Bexley Heath.

### THE EDITOR'S LETTER-BOX.

### *EMPLOYMENT AND TRAINING.*

### Laboratory Assistant.

IT all depends upon what your attainments are. If you are an expert bacteriologist there should no difficulty in obtaining an appointment as an assistant in a London research laboratory, provided your credentials from your teachers are satisfactory. In any case there is no harm in asking, and we suggest that you should apply to the Director of Research Laboratories, either at St. Mary's Hospital, the London Hospital, the Middlesex Hospital, or the Cancer Hospital.—" B. A."

### Paying Probationers.

WE are not aware that any convalescent institutions make a practice of taking paying probationers. We give you the names of several Homes for Children in the South of England, as requested, with a view to your making application, and it is possible that you may be able to enter into an arrangement with one of them which will meet your wishes: The Yarrow Convalescent Home for Children, Broadstairs, the Children's Branch of the Metropolitan Convalescent Institution, Broadstairs, Children's Seaside Home, The Point, Exmouth, Home for Convalescent Children, 33 Auckland Road, Southsea.— " G. M."

### *ACKNOWLEDGMENTS.*

**Bermondsey.**—" No Prayers, No Medicine," "The Organiser of the Blacklegs."—If our correspondent will send a letter containing the facts and sign it, it shall be treated on its merits. —ED. THE HOSPITAL.

***Sister H.*** — We thank you for your post-card, we fear that you do not fully understand our method of bringing patients into touch with our Approved Homes. In addition to maintaining a permanent register, easily accessible to patients on application, we announce the needs of patients in these columns from time to time, and we undertake to forward all communications received from the Homes, for which the yearly forwarding fee of 5s. is charged. It will thus be clear to you that it depends upon your promptness in replying to these announcements as to whether you derive the fullest benefit of registration.

**S. H.**—We are glad to know that our communication reached you, notwithstanding your oversight, and that the offer made you through the medium of the Bureau is likely to suit you.

**C. P. M.**— We have forwarded you further communications in response to your offer to phthisical nurses, and we hope to hear that you have been able to make a selection.

*Communications have been received and attended to from Amy Massie, Mabel A. Shoosmith, and Miss Sheldrake.*

### *MISCELLANEOUS.*

### Bed Inclinators.

THE choice of a bed inclinator depends upon the price which you are prepared to pay and the use to which you desire to put it. A useful appliance, which takes the place of the old-fashioned wooden blocks, is made by Messrs. J. Nesbit-Evans and Co., of Birmingham, and would probably serve your purpose. This is a stand constructed of steel tubing with rests arranged at three different heights, upon which the cross-bar, either at the head or the foot of the bedstead, can be placed. The price is about 18s. An excellent inclinator, which obviates the danger and difficulty of tilting bedsteads to the high inclination required for certain cases, can be obtained from Mr. Skeffington, of Blackheath, London, S.E. The apparatus consists of a light steel mattress frame which is placed in position by sliding it beneath the bedding; by means of pulley tackle attached to a hook in the wall or a head pole, and either to the foot corners or head corners of the mattress, as desired, the patient can be tilted to the inclination required.— " W. H."

### Balcony Bedsteads.

As an alternative to the use of bed trolleys, descriptions of which we gave you in our last issue, you can obtain a special balcony wheeling-out bedstead, equipped with a permanent wheeling fixture at the foot. The back legs of this bedstead are mounted on large castors, and by means of the attachment at the foot, which is actuated by a lever, the front legs can be raised from the floor. The bed can thus be readily transferred from place to place when desired. It is manufactured by Messrs. J. Nesbit-Evans and Co., of Birmingham, and is in use at the Glasgow Western Infirmary and other large hospitals, where it is found to be quite satisfactory. The special castors and attachment increase the price over that of the ordinary bedstead by about 14s. In purchasing new bedsteads this pattern is well worth consideration, but it would be unwise to discard serviceable bedsteads when the use of an inexpensive trolley, such as we suggested, involving a little additional labour, would meet your requirements.—" HOSPITAL SECRETARY."

**The Editor is prepared to make known without charge the needs of any who may be sick or in difficulties, and to guide them in making choice of Homes for treatment or convalescence. Reduced terms can often be arranged with Homes on the Approved List for those unable to pay full fees. No Home is included in the Approved List without medical and other testimony to its good management. Queries are specially invited from those who are engaged in any kind of philanthropical work. A new edition of the leaflet describing the purposes of the Bureau of Information is now ready and will be sent free of charge on application. For Rules for Correspondents see page 1.**

### Editor's Notices.

**Contributions:**

Contributions should be written, or preferably typed, on one side of the paper only, and all articles sent in are accepted upon the distinct understanding that they are forwarded to THE HOSPITAL only.

The Editor cannot undertake to return MSS. not used, but when a stamped directed envelope is enclosed the MSS. may be returned if a special request is made.

Accepted articles and paragraphs of news will be paid for after publication at the scale rate.

**Address:**

To prevent delay all contributions and letters on editorial business must be addressed exclusively to the Editor, "The Hospital," 29 Southampton Street, Strand, London, W.C. It is important that this regulation shall be strictly observed.

**Correspondence:**

Correspondence on all subjects is invited. The name and address of correspondents must be given as a guarantee of good faith, but not necessarily for publication.

**Special Articles:**

Special articles are invited, and questions, inquiries, and paragraphs upon all matters relating to the work, administration and management of general, special, mental, fever, cottage and convalescent hospitals, sanatoria, homes, institutions, societies and organisations for the treatment or care of the sick, injured and dependants of all classes. Every matter affecting the interests and welfare of the staff of all grades working in these institutions will receive special consideration.

**Administrative Medicine, Research and Items of News:**

Special terms will be paid for approved articles on subjects in this wide field by experts who have made some section of it their special study and interest. We wish to give prominence to every movement tending to advance accuracy of diagnosis, efficiency in treatment, and the development of modern methods to eradicate disease and promote the welfare of the suffering.

**A Bureau of Information:**

We invite inquiries and applications for information and help in providing for that numerous class of sufferers who require special care or house-room which they cannot obtain in their own homes or secure for themselves. We cannot, however, prescribe, or recommend practitioners

**Books for Review:**

Publishers are particularly requested to send advance proofs of any new books of importance whenever possible, as the Editor has made arrangements to publish immediate reviews on a new plan.

**Photographs, Plans, Blocks, and Illustrations:**

It is requested that wherever possible MSS. may be accompanied by illustrations in any of the above forms. The name of the sender and of the article to which it belongs should be written on the back of each photograph, drawing, or block for purposes of identification.

**Local Papers:**

Newspapers containing reports or news paragraphs should be marked and addressed to the Sub-Editor.

**The Coupon System:**

A coupon will be found at the bottom of the third inside page of the cover each week. This coupon must be attached to every question or inquiry to which an answer is desired in THE HOSPITAL.

### Unique Advantages.

FOR our readers who may be seeking a post in any department of Institutional Life special facilities are provided in this section of THE HOSPITAL. Every week a column is reserved for announcements of Appointments Wanted, which enables the worker to bring his or her needs directly to the notice of the Executive Officials of all classes of Institutions throughout the Kingdom as well as those responsible for Poor-Law and Municipal Administration. No other Journal possesses such a wide and comprehensive circulation in these directions, and THE HOSPITAL has proved a pre-eminently successful Medium for securing Appointments in every branch of the Institutional, Health and Sanitary Services. The charge for announcements in the Appointments Wanted column is 1s. for twenty-one words; and the form below is for the convenience of those readers who desire to avail themselves of the unique advantages which "The Institutional Worker" affords; when filled up it should be posted to

*The Manager*, THE HOSPITAL,
28 and 29 Southampton Street,
Strand, London, W.C.

Appointments Wanted, 21 words ... 1s. 0d.

| | | |
|---|---|---|
| | | |
| | | |
| | | |
| | | |
| | | |
| | | |

### Manager's Notices.

**Letters relating to the publication, sale, and advertising departments of "The Hospital" must be addressed to The Manager, "The Hospital," The Hospital Building, 28 and 29 Southampton Street, London, W.C.**

**Subscriptions** may begin at any time, and are payable in advance. Cheques and Post-Office Orders should be crossed London County and Westminster Bank, Covent Garden Branch, and made payable to the Manager of THE HOSPITAL.

RATES OF SUBSCRIPTION (including postage).

| *United Kingdom.* | | *Foreign and Colonial.* | |
|---|---|---|---|
| Three Months ... ... | 2s. 0d. | Three Months ... ... | 2s. 6d. |
| Six Months ... ... | 4s 0d | Six Months ... ... | 5s. 0d. |
| Twelve Months ... ... | 6s. 6d | Twelve Months ... ... | 8s. 6d. |

**Advertisements:**

To ensure insertion, all advertisements for THE HOSPITAL must reach the Manager not later than Wednesday morning in each week. For scale of charges see pages v and 4.

**Remittances:**

**It is especially requested that remittances be made by Cheque or Postal Order, and in halfpenny stamps only when the amount is under sixpence.**

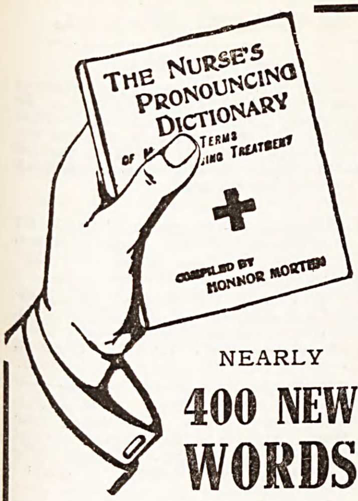

NEARLY

**400 NEW WORDS**

have been added to the

**NEW EDITION**

of

### The Nurse's Pronouncing Dictionary

### OF MEDICAL TERMS AND NURSING TREATMENT

Compiled by **HONNOR MORTEN.**

Eighth Edition, thoroughly Revised and much Enlarged.

Edited by **MARY I. BURDETT.**

The new issue of this well-known Dictionary is the **most complete work** of the kind published. It has been completely revised and some hundreds of new words have been added, consequently the utmost reliance can be placed in the information it contains.

To be up-to-date you should GET A COPY TO-DAY.

**Price, in cloth, 2/- post free; in leather, 2/6 net, by post 2/8.**

*Of all Booksellers, or of—*

**The Scientific Press, Ld.**

(*The Nurses' Publishers*),

28/29 Southampton Street, STRAND, LONDON, W.C.

### HANDBOOKS

### For TRAINED WORKERS

POCKET·GUIDE SP SERIES

**1/- net.** **1/1 Post free.**

**Bound in Limp Cloth.**
**Suitable Size for the Pocket.**

The works which comprise the S.P. Pocket Guide Series are intended for ready reference, consequently the aim of the Publishers has been to make them as concise and lucid as possible. Each book is written by an expert on the subject of which it treats, and the utmost reliance therefore can be placed in the information it imparts.

**Essentials of Fever Nursing.** By LYTTON MAITLAND, M.D. (Lond.), M.B., B.S., D.P.H. (Camb.).

**Manual and Atlas of Swedish Exercises.** *(With over 60 Illustrations.)* By THOMAS D. LUKE, M.D., F.R.C.S.

**Bandaging Made Easy.** *(Illustrated with over 90 Diagrams.)* By M. HOSKING, Sister-in-Charge, Tredegar House, Bow, E.

**How to Write and Read Prescriptions.** By LYTTON MAITLAND, M.D. (Lond.), M.B., B.S., D.P.H. (Camb.).

**Principal Drugs and their Uses.** By A PHARMACIST.

**Asepsis and How to Secure It.** By H. W. CARSON, F.R.C.S.

**Treatment after Operations.** *Rules for Nursing after General and Special Operations.* By MARY WILES.

**The Nurse's Duties before and during Operations.** By MARGARET FOX, Matron, Prince of Wales's General Hospital, Tottenham.

**Other works, in amplification of this Series, will be announced in due course.**

*Published by*

**The SCIENTIFIC PRESS, Ltd.**

28/29 Southampton Street, STRAND, LONDON, W.C.

By Post **5/4**

**It is possible to secure "the most up-to-date and Complete Guide to Nursing that has yet been written."**

*Vide THE NURSING MIRROR.*

### A HANDBOOK FOR NURSES

**With Examination Questions based upon the contents of the Chapters.**

**By J. K. WATSON, M.D.Edin., C.M.**

**5th Edition, Entirely Revised, and Enlarged to 544 pages**

The title of this book very inadequately describes its scope. It would be more accurately entitled "*THE* Handbook for Nurses," since the information it contains renders it of real and genuine service to the modern Nurse. The fact that **31,500** copies of former editions have been sold is indisputable evidence of its value. The work forms practically an Encyclopædia of Medical, Surgical and Monthly Nursing, and there are few, if any, Nursing details that do not find representation in its pages. If you have not seen the New Edition we would strongly advise you to take the earliest opportunity of doing so.

It is obtainable of all Booksellers, or direct from . . . .

**THE NURSES' PUBLISHERS,**

**28/29 SOUTHAMPTON STREET, STRAND - LONDON, W.C.**

